# Calorie seeking, but not hedonic response, contributes to hyperphagia in a mouse model for Prader–Willi syndrome

**DOI:** 10.1111/ejn.12972

**Published:** 2015-06-25

**Authors:** Jennifer R. Davies, Trevor Humby, Dominic M. Dwyer, Alastair S. Garfield, Hannah Furby, Lawrence S. Wilkinson, Timothy Wells, Anthony R. Isles

**Affiliations:** ^1^ Behavioural Genetics GroupMRC Centre for Neuropsychiatric Genetics and GenomicsNeuroscience and Mental Health Research InstituteCardiff UniversityHadyn Ellis BuildingCardiffCF24 4HQUK; ^2^ School of MedicineCardiff UniversityCardiffUK; ^3^ School of PsychologyCardiff UniversityCardiffUK; ^4^ School of PsychologyUniversity of New South WalesSydneyNSWAustralia; ^5^ Centre for Integrative PhysiologyUniversity of EdinburghEdinburghUK; ^6^ School of BiosciencesCardiff UniversityCardiffUK

**Keywords:** hyperphagia, lick‐cluster analysis, palatability, Prader–Willi syndrome

## Abstract

Prader–Willi syndrome (PWS) is a neurodevelopmental disorder caused by deletion or inactivation of paternally expressed imprinted genes on human chromosome 15q11‐q13, the most recognised feature of which is hyperphagia. This is thought to arise as a consequence of abnormalities in both the physiological drive for food and the rewarding properties of food. Although a number of mouse models for PWS exist, the underlying variables dictating maladaptive feeding remain unknown. Here, feeding behaviour in a mouse model in which the imprinting centre (IC) of the syntenic PWS interval has been deleted (PWS ^*ICdel*^ mice) is characterised. It is demonstrated that PWS ^*ICdel*^ mice show hyperghrelinaemia and increased consumption of food both following overnight fasting and when made more palatable with sucrose. However, hyperphagia in PWS ^*ICdel*^ mice was not accompanied by any changes in reactivity to the hedonic properties of palatable food (sucrose or saccharin), as measured by lick‐cluster size. Nevertheless, overall consumption by PWS ^*ICdel*^ mice for non‐caloric saccharin in the licking test was significantly reduced. Combined with converging findings from a continuous reinforcement schedule, these data indicate that PWS ^*ICdel*^ mice show a marked heightened sensitivity to the calorific value of food. Overall, these data indicate that any impact of the rewarding properties of food on the hyperphagia seen in PWS ^*ICdel*^ mice is driven primarily by calorie content and is unlikely to involve hedonic processes. This has important implications for understanding the neural systems underlying the feeding phenotype of PWS and the contribution of imprinted genes to abnormal feeding behaviour more generally.

## Introduction

Prader–Willi syndrome (PWS) is a neurodevelopmental disorder caused by deletion or inactivation of paternally expressed imprinted genes on human chromosome 15q11‐q13 (Cassidy *et al*., 2000). Imprinted genes are those that are epigenetically marked in a parent‐of‐origin‐dependent manner during gametogenesis, and consequently expression of these genes in somatic cells is from one parental allele only. Therefore, mutations leading to deletion or inactivation of the normally paternally expressed genes within the 15q11‐q13 imprinting cluster results in complete ablation of these gene products and PWS.

Individuals with PWS are characterised by short stature, infantile hypotonia, hypogonadism, cognitive disabilities and behaviour problems, including stubbornness, obsessive‐compulsive behaviours and skin picking (Goldstone, 2004). However, the most recognised features of the syndrome are hyperphagia and a preoccupation with food (Chen *et al*., 2007), which have historically been considered as occurring in two stages. During the neonatal period, a poor ability to suckle and a failure to thrive is accompanied by a lack of weight gain, despite normal calorie intake (Stage 1). However, by 2–4 years old, abnormal eating behaviour of children with PWS is manifested by delayed satiety, premature return of hunger after eating a meal, the seeking and hoarding of food and food‐related objects, and even the ingestion of non‐food items (Stage 2). Recently, Miller *et al*. (2011) have identified up to seven distinct nutritional stages and transitional periods that reflect different phases of food intake, underlying neuroendocrine status, and degree of obesity in individuals with PWS highlighting the complexity of the phenotype.

The later PWS hyperphagia symptoms result in an increased likelihood of morbid obesity and, without careful control of food intake and the food environment, death from obesity‐related complications. Individuals with PWS consume up to three times the normal caloric intake at a given meal (Holland *et al*., 1993, 1995), and stomach rupture from extreme overeating has been reported (Wharton *et al*., 1997). Approximately one‐third of the PWS population maintains > 200% of their ideal body weight (Bray *et al*., 1983). Nonetheless, despite the central importance of hyperphagia in PWS, the neural mechanisms driving this feeding behaviour are not fully understood.

The neural circuitry controlling feeding behaviour in mammals is complex, but can be broadly divided into two parallel systems that interact to influence food intake (Hommel *et al*., 2006; Lutter & Nestler, 2009). The homeostatic system comprises hormonal regulators of hunger, satiety and adiposity levels, which act on hypothalamic and brainstem circuits to stimulate or inhibit feeding in order to maintain appropriate levels of energy balance. The lack of a satiety response seen in patients with PWS (Lindgren *et al*., 2000) is thought to be associated with hypothalamic dysfunction and, in part at least, with levels of ghrelin. Ghrelin is a gut‐derived peptide hormone that signals to the hypothalamus and other brain regions, promoting feeding and adiposity (Wells, 2009), and circulating levels are greatly elevated in PWS (Cummings *et al*., 2002; Haqq *et al*., 2003; Goldstone, 2004).

However, appetite can also be driven by factors other than physiological needs, with the reward system of the brain also playing an important role in initiating and maintaining feeding behaviour (Volkow *et al*., 2011). In addition to increased activation in the hypothalamus of individuals with PWS during fasting (Holsen *et al*., 2006; Dimitropoulos & Schultz, 2008) and following food intake (Shapira *et al*., 2005), neuroimaging studies have also implicated reward‐associated areas of the brain. For example, post‐meal hyperactivation has been seen in response to various food stimuli in the nucleus accumbens (Shapira *et al*., 2005), amygdala, hippocampus (Holsen *et al*., 2006), medial prefrontal cortex (Miller *et al*., 2007) and the orbital frontal cortex (Hinton *et al*., 2006; Holsen *et al*., 2006), suggesting a dysfunction in reward and satiety circuitry. More recently, alterations in functional connectivity in brain regions implicated in both eating and reward‐related processing in individuals with PWS have been shown (Zhang *et al*., 2013). However, this aspect of feeding behaviour in PWS is less well understood, and the neural bases unclear.

Whilst not completely homologous, the degree of synteny between the mouse and human PWS gene interval has allowed genetic models for PWS to be generated and behaviourally characterised (Relkovic & Isles, 2013). Nevertheless, although a number of these mouse models demonstrate abnormal feeding behaviour and/or hyperphagia (Bischof *et al*., 2007; Ding *et al*., 2008), the underlying controlling variables dictating maladaptive feeding remain unknown. To address this issue, feeding behaviour in a mouse model in which the imprinting centre (IC) of the syntenic PWS interval has been deleted (PWS^*ICdel*^ mice) has been characterised. It has been demonstrated that PWS^*ICdel*^ mice have increased levels of circulating ghrelin and consume more food following overnight fasting, and when more palatable with sucrose. However, the hyperphagia was not accompanied by any evidence of changes in reactivity to the hedonic (pleasurable) properties of food, insofar as PWS^*ICdel*^ mice did not show an enhanced hedonic impact of palatable food (sucrose) measured by lick‐cluster analysis (LCA; Dwyer, 2012). Instead, LCA responses to non‐caloric saccharin combined with converging findings from a continuous reinforcement schedule (CRF) with saccharin reward indicate that PWS^*ICdel*^ mice show a marked heightened sensitivity to the calorific value of food. Taken together, these data indicate that the hyperphagia seen in PWS^*ICdel*^ mice is primarily due to dysfunction of the homeostatic system, and that any impact on the rewarding properties of food consumption are unlikely to involve hedonic processes. This has important implications for understanding of the neural systems underlying the feeding phenotype of PWS and the contribution of imprinted genes to abnormal feeding behaviour more generally.

## Materials and methods

### Subjects

PWS^*ICdel*^ (specifically, PWS^m+/*ICdel*^) and wild‐type (WT) adult mice were maintained on an outbred CD1 background, and experimental cohorts bred as described previously (Doe *et al*., 2009; Relkovic *et al*., 2010). Litters weaned together into groups of two‐five subjects per cage. All subjects were housed under standard temperature‐ and humidity‐controlled conditions, with a 12‐h light : 12‐h dark cycle. All experiments and measurements were taken during the light phase. For the LCA and continuous reinforcement task (CRT) experiments, all subjects had *ad libitum* access to water, but home cage food was restricted to 8 h access/day. This regime maintained the subjects at ~90% of free‐feeding body weight. All procedures were conducted in accordance with the requirements of the UK Animals (Scientific Procedures) Act 1986, under the remit of Home Office licence number 30/2673. These procedures were also approved by the appropriate ethics committee at Cardiff University.

### Circulating ghrelin

Twelve (six female, six male) WT and 12 (six female, six male) PWS^*ICdel*^ mice were tested for basal levels of circulating ghrelin. Animals were killed by cervical dislocation, blood samples were obtained by cardiac puncture using BD microtainers (SST Amber Tubes, BD Biosciences, UK), and aliquots of separated plasma were stored at −20 °C. Plasma ghrelin (total) concentration was determined by RIA (Millipore/Linco, St Charles, MO, USA).

### Food consumption

Consumption of wet mash, consisting of 1 part standard diet and 1 part water, was measured in male and female WT and PWS^*ICdel*^ mice (4–6 months old). Additionally, in a one‐off experiment, consumption of wet mash plus 20% sucrose (w/w) was measured. Subjects were tested individually, outside of the home cage but in standard, equivalent‐sized shoe‐box cages under low lighting levels. A pot of pre‐weighed wet mash was placed in the test cage and the mice were allowed to consume freely for 30 min. Afterwards, the pot was re‐weighed, the difference in weight equalling the amount of food consumed.

Sixteen (eight female, eight male) WT and 12 (six female, six male) PWS^*ICdel*^ mice were habituated to the procedure for 2 days prior to the onset of testing, using basic wet mash. The following tests were conducted: consumption of wet mash containing 20% sucrose following normal prior access to food in the home cage (‘20% sucrose’); and consumption of wet mash following 16 h overnight (17:00–09:00 h) fasting (all food removed from home cages, water was provided *ad libitum*; ‘O/N fasting’). All testing took place between 09:00 h and 11:00 h.

### LCA

Training and testing took place in a separate experimental room. Mice were trained and tested in 16 custom‐made drinking chambers (Med Associated, St Albans, VT, USA). These were 32 × 15 × 12 cm (L × W × H), with steel mesh flooring and white acrylic walls. Subjects were placed individually into the test cages and given 30 min to drink the available solutions through drinking spouts made of stainless‐steel attached to 50 mL cylinders. A contact‐sensitive lickometer registered the time of each lick to the nearest 0.01 s, and a microcomputer running med‐pc software (Med Associates) controlled the equipment and recorded the data. A lick cluster was defined as a series of licks where the inter‐lick interval (ILI) did not exceed 500 ms, therefore any lick occurring > 500 ms after the previous would be recorded in a new lick cluster (Davis & Smith, 1992). Subject data where the number of total licks were < 40 were omitted. Test solutions were 2%, 8% and 16% sucrose and 0.1% saccharin (all wt/v) made up with tap water.

#### Experiment 1

Hedonic reactions to increasing concentrations of sucrose (2%, 8% and 16% sucrose) were assessed in 10 (five female, five male) PWS^*ICdel*^ and 23 (13 female, 10 male) WT mice. Animals were given eight sessions of 8% sucrose, with a single session each day, to habituate to the test environment and learn to drink from the lickometer apparatus. The test concentrations were 2% and 16% sucrose with the order of presentations counterbalanced across genotype and sex. No order effect was found (main effect or interaction with genotype), so groups were pooled. Animals received five sessions at each test concentration. Performance across the last three sessions (generally the most consistent consecutive sessions) at each concentration was averaged for analysis.

#### Experiment 2

To assess the effect of calories on palatability and consummatory behaviour, sucrose and saccharin were compared in a separate group of 11 (five female, six male) PWS^*ICdel*^ and 12 (10 female, two male) WT animals. Animals were given seven sessions of 8% sucrose and seven sessions of 0.1% saccharin (Sigma, UK), with a single session each day. Testing was counterbalanced for reinforcer, genotype and sex. No order effect was found (main effect or interaction with genotype), so groups were pooled. Performance across the three most consistent consecutive sessions at each concentration was averaged for analysis.

### CRT

Eight (four female, four male) PWS^*ICdel*^ and 11 (three female, eight female) WT animals underwent an instrumental task in nine‐hole boxes (Campden Instruments, UK; Humby *et al*., 1999) using a CRF. Boxes were configured such that only the central aperture of the response array (10 mm diameter, 10 mm from the chamber floor) was available. Stimuli presentations and subject responses were controlled/recorded by custom‐written software programmes (Arachnid).

For training, mice were habituated to the test chambers for six 20‐min sessions (one session per day) in which 20 μL of reinforcer was presented on a VI30 schedule, with the food magazine illuminated from the time of delivery until the mouse was recorded as collecting the reinforcer. The nose‐poke aperture was blocked for these initial training sessions. For the first three sessions, the door to the food magazine was wedged open so that the mice could gain access to the reinforcer easily, but for the remaining three sessions mice were required to push the door open.

Following magazine training, the mice undertook sessions under a CRF (single nose‐poke to initiate delivery of the reward (~22 μL, 8% sucrose), this continued until each animal reached a stable performance (> 80 rewards received per session for three consecutive days). Animals were then switched to 0.1% sodium saccharin (Sigma, UK) as a reinforcer for three sessions, followed by a further three sessions with 8% sucrose. Animals were also examined for their response to water. Following three consecutive days of stable performance (> 80 rewards received per session) with 8% sucrose, animals were switched to receiving water alone as a reinforcer. All CRF sessions terminated after collection of 100 rewards or if 30 min had elapsed (whatever occurred first). The last three sessions for each reinforcer were analysed.

### Data analysis

All data were analysed using SPSS 20 (SPSS, USA). Data were analysed by Student's *t*‐test or mixed anova, with between‐subjects factors of GENOTYPE (PWS^*ICdel*^ vs. WT) and SEX (male or female); and within‐subject factors as appropriate (i.e. time, session, reward type, reward concentration). All significance tests were performed at alpha level of 0.05 and, where significant interactions were identified in the main anova,* post hoc* tests using appropriate pair‐wise comparisons were performed. For repeated‐measures analyses, Mauchly's test of sphericity of the covariance matrix was applied. Greenhouse–Geisser corrections were applied as necessary, and adjusted degrees of freedom are provided.

Where main effects of SEX and/or interactions between SEX and GENOTYPE occur, male and female data are reported separately. However, for the majority of data no sex differences were found and the data presented are pooled.

## Results

### Hyperghrelinaemia and increased food consumption in PWS^*ICdel*^ mice

**Figure 1:**
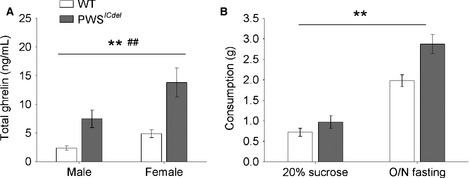
Prader–Willi syndrome (PWS)^*ICdel*^ mice show hyperghrelinaemia and increased food intake under basal and post‐fast conditions, and with free sucrose consumption. (A) Circulating plasma ghrelin levels are elevated in PWS ^*ICdel*^ mice relative to controls. (B) Analysis of free consumption of standard food (as wet mash) for 30 min. PWS ^*ICdel*^ mice consumed significantly more food containing 20% sucrose than wild‐type (WT) littermates. Similarly, PWS ^*ICdel*^ mice consumed significantly more food (basic wet mash) than WT littermates following overnight fasting. Data shown are mean ± SEM (GENOTYPE, **P < 0.01; SEX, ^##^ *P* < 0.01).

Circulating ghrelin was elevated 2.8‐ to threefold in *ad libitum*‐fed PWS^*ICdel*^ mice (Fig. 1A; main effect of GENOTYPE, *F*_1,23_ = 21.07, *P* < 0.001). As expected, ghrelin levels were increased, by ~1.8‐fold, in females compared with males (main effect of SEX, *F*_1,23_ = 8.37, *P* = 0.009). However, there was no interaction between GENOTYPE and SEX on ghrelin measures (*F*_1,23_ = 1.59, *P* = 0.22).

PWS^*ICdel*^ mice consumed more food under both 20% sucrose and fasted conditions (Fig. 1B; main effect of GENOTYPE, *F*_1,24_ = 16.36, *P* < 0.001). There was no main effect of SEX on consumption (*F*_1,24_ = 1.88, *P* = 0.18), and no interaction between GENOTYPE and SEX (*F*_1,24_ = 3.08, *P* = 0.092); consequently pooled data are presented.

### LCA

#### Experiment 1: hedonic reaction to palatable food is not enhanced in PWS^*ICdel*^ mice

**Figure 2:**
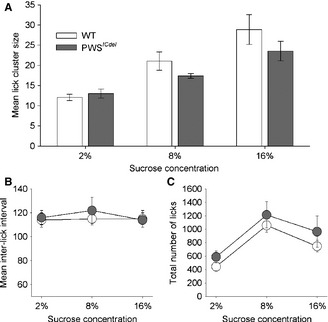
Lick‐cluster analysis (LCA) showing hedonic response to increasing sucrose concentration in wild‐type (WT) and Prader–Willi syndrome (PWS)^*ICdel*^ mice. (A) Lick‐cluster size (LCS) increases with increasing sucrose concentration, showing a linear relationship between this hedonic measure and palatability. However, there was no statistical different between WT and PWS ^*ICdel*^ mice in LCS measures across all sucrose concentrations. Similarly, (B) inter‐lick interval (ILI) and (C) total number of licks were also equivalent in WT and PWS ^*ICdel*^ mice. Data shown are mean ± SEM.

The enhanced consumption of food could be due to an abnormal hedonic reaction to food in the PWS^*ICdel*^ mice. In order to test this directly, LCA was used. Lick‐cluster size (LCS), the main hedonic measure, displayed the typical positive relationship with increasing concentration of sucrose (Fig. 2A; main effect of CONC, *F*_1.4,44.4_ = 8.82, *P* = 0.002). However, there was no difference in LCS between PWS^*ICdel*^ and WT mice (main effect of GENOTYPE, *F*_1,31_ = 0.63, *P* = 0.433), and no interaction between CONCENTRATION and GENOTYPE (*F*_1.4,44.4_ = 0.65, *P* = 0.477). Additionally, there was no genotype differences for the average ILI at all concentrations (Fig. 2B; main effect of GENOTYPE, *F*_1,31_ = 1.64, *P* = 0.21). ILI reflects the physical aspects of consumption, such as postural or motor problems, which may possibly confound the LCS data. Consumption of sucrose, as measured by total number of licks, also increased with CONCENTRATION (Fig. 2C; *F*_2,31_ = 23.83, *P* < 0.001). Whilst the PWS^*ICdel*^ mice had numerically higher total lick numbers, this was not statistically significant (main effect of GENOTYPE, *F*_1,31_ = 1.494, *P* = 0.23).

There were no main effects of SEX on any of the measures (LCS, *P* = 0.205; ILI, *P* = 0.092; total licks, *P* = 0.247), nor were there any interactions between SEX and GENOTYPE (LCS, *P* = 0.365; ILI, *P* = 0.674; total licks, *P* = 0.149).

#### Experiment 2: lack of nutritional value causes reduced avidity for saccharin in PWS^*ICdel*^ mice

**Figure 3:**
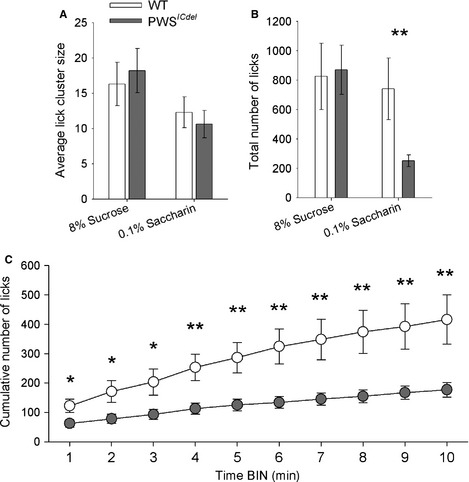
Lick‐cluster analysis (LCA) showing hedonic response to caloric (8% sucrose) and non‐caloric (0.1% saccharin) tastants in wild‐type (WT) and Prader–Willi syndrome (PWS)^*ICdel*^ mice. (A) Although there is a general difference between sucrose and saccharin in lick‐cluster size (LCS), again there is no difference between WT and PWS ^*ICdel*^ mice, suggesting that perceived palatability is the same for both genotypes. (B) However, consumption of saccharin, as measured by total number of licks, is significantly reduced in PWS ^*ICdel*^ mice and WT controls. There is no difference between WT and PWS ^*ICdel*^ mice in total number of licks with 8% sucrose. (C) Analysis of cumulative lick data binned across the first 10 min of testing revealed that this reduced motivation for saccharin seen in PWS ^*ICdel*^ mice is present throughout the duration of the session (asterisks relate to *post hoc* comparisons). Data shown are mean ± SEM (GENOTYPE, *P < 0.05, **P < 0.01).

In order to dissociate the impact of nutrition and taste, 8% sucrose, which has both nutritional and taste value, was compared with the calorie‐free artificial sweetener saccharin, which has taste value alone. For LCS (Fig. 3A; *F*_1,21_ = 5.38, *P* = 0.034) and total number of licks (Fig. 3B; *F*_1,21_ = 5.14, *P* = 0.031), there was a general main effect of SOLUTION, with both lower for 0.1% saccharin than 8% sucrose. Again, there was no significant main effect of GENOTYPE for LCS (*F*_1,21_ = 0.49, *P* = 0.491), and no interaction between GENOTYPE and SOLUTION (*F*_1,21_ = 0.134, *P* = 0.782). The LCS data suggest no difference in the hedonic value or taste of saccharin between the PWS^*ICdel*^ and WT mice. However, there was an interaction between GENOTYPE and SOLUTION for the total number of licks (*F*_1,21_ = 4.626, *P* = 0.042). *Post hoc* tests demonstrated that there was a threefold reduction in total number of licks in PWS^*ICdel*^ mice with saccharin (*t* = 2.31, *P* < 0.040). As previously, there was no statistical difference in total licks for 8% sucrose between the PWS^*ICdel*^ and WT mice (*t* = 0.40, *P* = 0.879). Binned repeated‐measure analysis of cumulative lick totals confirmed this difference between PWS^*ICdel*^ and WT mice saccharin session (Fig. 3C; main effect of GENOTYPE, *F*_1,20_ = 8.98, *P* = 0.007). Furthermore, although there was a significant interaction between GENOTYPE and BIN (*F*_9,180_ = 7.472, *P* < 0.001), *post hoc* analysis confirmed the GENOTYPE difference was present in the first minute of testing (*t* = 2.171, *P* = 0.042).

There were no main effects of SEX on any of the measures (LCS, *P* = 0.973; total licks, *P* = 0.173; cumulative licks, *P* = 0.382). Nor were there any interactions between SEX and GENOTYPE (LCS, *P* = 0.715; total licks, *P* = 0.283; cumulative licks, *P* = 0.279).

### CRT

#### PWS^*ICdel*^ mice display apathy for non‐caloric reinforce

**Figure 4:**
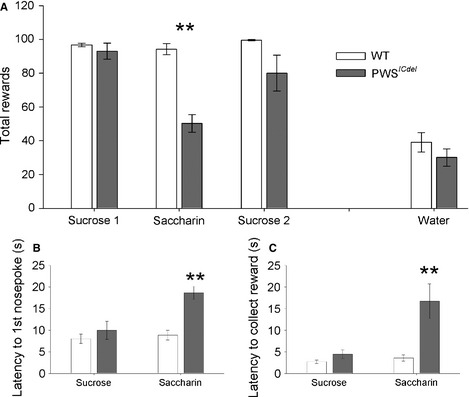
Responding in a continuous reinforcement task (CRT) demonstrates reduced consumption and interest in saccharin in Prader–Willi syndrome (PWS)^*ICdel*^ mice relative to wild‐type (WT) controls. (A) The total number of sucrose rewards received is the same in PWS ^*ICdel*^ and WT mice, both before and after experience of saccharin. However, PWS ^*ICdel*^ mice show a significant reduction in the number of saccharin rewards received relative to WT mice, which exhibit similar levels to 8% sucrose. Both genotypes show a reduced, but equivalent, level of responding to water. The altered motivation of PWS ^*ICdel*^ mice for saccharin is further reflected in latency measures (B: latency to first nose‐poke; C: average latency to collect rewards), which are equivalent to WT mice for 8% sucrose, but significantly increased with 0.1% saccharin. Data shown are mean ± SEM (GENOTYPE, **P < 0.01).

In order to investigate the decreased motivation of the PWS^*ICdel*^ mice in saccharin more readily, first, animals were tested using an 8% sucrose reinforcer under a CRF. Both PWS^*ICdel*^ and WT mice readily achieved high levels of performance, collecting > 90 rewards per session, and often reaching the ceiling of 100 rewards within the session duration. There was no significant difference between PWS^*ICdel*^ and WT mice in the total number of 8% sucrose rewards earned (Fig. 4A; *t* = 0.87, *P* = 0.400). A similar performance level was also observed between genotypes for other parameters measured with no significant difference in latency to make the first nose‐poke for sucrose reward (Fig. 4B; *t* = 0.91, *P* = 0.378) and latency to collect the sucrose reward (Fig. 4C; *t* = 1.59, *P* = 0.147).

Switching from 8% sucrose to 0.1% saccharin produced a general reduction in responding (Fig. 4A; main effect of SOLUTION, *F*_1,17_ = 30.15, *P* < 0.001). There was also a main effect of GENOTYPE (*F*_1,17_ = 63.96, *P* < 0.001). However, mirroring the pattern seen in the LCA, there was a significant interaction between SOLUTION × GENOTYPE interaction (Fig. 4A; *F*_1,17_ = 24.16, *P* < 0.001), with *post hoc* testing demonstrating that PWS^*ICdel*^ and WT made an equal number of responses with 8% sucrose (*t* = 0.87, *P* = 0.400), but that PWS^*ICdel*^ mice demonstrated a significant 50% reduction in responding to 0.1% saccharin (Fig. 4A; *t* = 7.66, *P* < 0.001). When mice were switched back to 8% sucrose, the performance of the PWS^*ICdel*^ mice returned to the same level as in the first CRF block, and was again not significantly different to that of the WT mice (Fig. 4A; Sucrose 2, *t* = 1.84, *P* = 0.109). The recovery of performance in the PWS mice when sucrose was reintroduced as the reward suggests that their behaviour for saccharin was not simply the product of failing to engage with the task. In addition to a reduced number of rewards received, PWS^*ICdel*^ mice also show changes in latency measures that reflect a reduced motivation for saccharin. These include an increased latency to make the first nose‐poke (Fig. 4B; *t* = 5.29, *P* < 0.001) and an increased latency to collect the reward (Fig. 4C; *t* = 3.21, *P* = 0.014).

Interestingly, the number of rewards earned by the PWS^*ICdel*^ for saccharin was similar to rewards earned for water (Fig. 4A) in both PWS^*ICdel*^ and WT mice. Comparing responding for saccharine and water directly supported this observation, with an interaction between SOLUTION and GENOTYPE (*F*_1,52_ = 8.89, *P* = 0.004). Although there were main effects of both SOLUTION (two‐way anova,* F*_1,52_ = 42.67, *P* < 0.001) and GENOTYPE (*F*_1,52_ = 20.49, *P* < 0.001), *post hoc* analysis revealed these were driven mainly by the difference between PWS^*ICdel*^ and WT mice in responding to saccharin, as no significant difference between groups was found for responding to water (*t* = 1.12, *P* = 0.273).

Again, there were no main effects of SEX on any of the measures (number of rewards, *P* = 0.388; latency to nose‐poke, *P* = 0.808; latency to collect reward, *P* = 0.768). Nor were there any interactions between SEX and GENOTYPE (number of rewards, *P* = 0.805; latency to nose‐poke, *P* = 0.901; latency to collect reward, *P* = 0.670).

## Discussion

In humans there is evidence that obese individuals prefer and consume high‐calorie palatable foods more than those of normal weight. Individuals with PWS also show a strong preference for high‐calorie fat or carbohydrate foods over foods with lower caloric values (Glover *et al*., 1996; Joseph *et al*., 2002). Given unrestricted access to food, PWS individuals will consume ~3 times more calories than that of individuals matched on age and body mass index, and eat for a longer period of time (Zipf & Berntson, 1987; Lindgren *et al*., 2000). Here, the aim was to examine in detail the food‐related behaviour in a mouse model for PWS, in order to gain insight into the psychological and physiological mediators of overeating in PWS. The PWS^*ICdel*^ mice were hyperghrelinergic and showed hyperphagia, consuming more food following overnight fasting, and also when tested with food made more palatable with the addition of sucrose. These latter data hint at a possible altered hedonic response to palatable foods. However, explicit tests of hedonic responses using LCA indicated no difference between PWS^*ICdel*^ and WT littermate controls in LCS to varying concentrations of sucrose (2%, 8% and 16%) and 0.1% saccharin. In contrast, overall consumption and motivation to work for calorie‐free saccharin was greatly reduced in PWS^*ICdel*^ mice in both the LCA and a CRT, despite no suggestion of such an effect with caloric sucrose solutions. Taken together, any impact of the rewarding properties of food on the hyperphagia seen in PWS^*ICdel*^ mice is driven primarily by calorie content and is unlikely to involve hedonic processes.

PWS behaviour is dominated by hyperphagia and insatiable appetite. The PWS^*ICdel*^ model used here recapitulates many aspects of typical PWS consummatory behaviour, including hyperphagia, which became more pronounced after overnight fasting. Furthermore, similar to patients with PWS (Goldstone, 2004) and other mouse models for PWS (Stefan *et al*., 2005; Ding *et al*., 2008), the PWS^*ICdel*^ mice were hyperghrelinergic. Whilst it has been suggested that increased ghrelin may not be causal to the abnormal eating patterns observed in PWS, but perhaps reflects a compensatory effect, elevated circulating levels of ghrelin may still underpin the hyperphagia/failed‐satiety response seen here, as well as that seen in patients with PWS (Purtell *et al*., 2011) and in other models (Bischof *et al*., 2007; Ding *et al*., 2008). This is further underlined by the fact that a *Snord116del* mouse model for PWS was less sensitive to the acute anorectic effects of ghrelin receptor antagonist and reverse agonist (Lin *et al*., 2014).

In addition to increased consumption of standard food following overnight fasting, a free consumption test also demonstrated that PWS^*ICdel*^ mice have an increase in consumption of sucrose‐containing food. This mirrors previous findings showing increased consumption and preferences for sucrose and condensed milk solutions (Doe *et al*., 2009; Relkovic *et al*., 2010, 2012). However, as a means of assaying reward‐related behaviour, consumption measures alone are confounded by several factors (Grill & Norgren, 1978; Davis, 1998), so in order to explicitly examine hedonic response in the PWS^*ICdel*^ mice, LCA was used, a well‐established rodent behavioural measure indexing ‘liking’ of food (Davis & Smith, 1992; Dwyer, 2012). When drinking, rodents do not lick continuously but perform repeated runs of licks (clusters) separated by pauses of varying length. These clusters are related to the nature of the solution being consumed and can be used as a reliable index of stimulus palatability and, hence, the affective component of reward value (Davis & Smith, 1992; Dwyer, 2012). This feature of behaviour was evident in the present studies, as in general LCS increased with increasing sucrose concentrations. However, when presented with a range of sucrose solutions there was no difference in LCS between PWS^*ICdel*^ and WT mice. Therefore, the data here and elsewhere (Doe *et al*., 2009; Relkovic *et al*., 2010, 2012) would suggest that whilst PWS^*ICdel*^ mice may consume more of a palatable food, this is not driven by an increased hedonic response. This conclusion is in contrast to inferences from previous neuroimaging studies with PWS subjects, which showed a hyper‐responsive reward circuit in relation to food generally (Miller *et al*., 2007) and in response to high‐calorie foods compared with controls (Dimitropoulos & Schultz, 2008). However, unlike the LCA used here, these studies cannot dissociate the individual aspects of reward, i.e. hedonic value, motivation and learned associative predictions. Also, the neural responses seen in functional magnetic resonance imaging were to food cues (e.g. pictures of food), not food receipt (e.g. tasting a milkshake), and therefore may reflect a measure of the anticipatory, rather than consummatory, phase of feeding.

Instead of palatability underlying food preference in PWS, a key factor may be the calorific value. To test the effect of calories, the response of PWS^*ICdel*^ mice to saccharin was examined, which is calorie free, in both the LCA and CRT. In both tests, consumption of saccharin was significantly reduced in PWS^*ICdel*^ mice compared with WT controls, as indexed by the total number of licks in the LCA and the total number of rewards obtained in the CRT. This lack of motivation for saccharin shown by PWS^*ICdel*^ mice was echoed in latency measures within the CRT, which are equivalent to WT controls when receiving sucrose but then increase significantly when receiving saccharin. Critically, however, this reduced consumption and interest was not due to the perceived taste of saccharin as, whilst there was an overall reduction in LCS for 0.1% saccharin in both PWS^*ICdel*^ and WT mice compared with 8% sucrose, there was no difference between groups. Nor was this behaviour simply due to a failure to engage following repeated task sessions; in the LCA the order of exposure was counter‐balanced and, in the CRT, reverting to 8% sucrose as a reinforcer returned PWS^*ICdel*^ performance to pre‐saccharin levels. Moreover, consumption of the dilute caloric solution of 2% sucrose was the same for PWS^*ICdel*^ and WT mice despite 2% sucrose and 0.1% saccharin eliciting equivalent LCS. These data indicate the difference in consumption of saccharin between PWS^*ICdel*^ and WT mice is not due to perceived palatability or hedonic value, but is specifically related to calorie content. Interestingly, WT mice are sensitive to calorific content, as when water is used as a reinforcer in the CRT, responding is reduced in both genotypes, and to equivalent levels as PWS^*ICdel*^ performance with saccharin. This could suggest that PWS^*ICdel*^ have a heightened motivational sensitivity to the calorific value of food and/or are more able to dissociate calorie content from taste.

Although considered to be a model of obesity, a more accurate description might be that PWS is in fact a model of starvation due to abnormal hypothalamic pathways incorrectly interpreting the absence of satiation as hunger (Holland *et al*., 2003). Such a perceived negative energy balance could explain a number of aspects of PWS, including hyperphagia and hypoactivity (Butler *et al*., 2007), both of which are also components of the PWS^*ICdel*^ phenotype (Relkovic *et al*., 2010), and also the increased adiposity but reduced lean mass (Theodoro *et al*., 2006). Similarly, although hunger following fasting in non‐obese healthy adults increases the reported appeal of both high‐calorie and low‐calorie foods, there is a specific enhanced activation of brain reward systems in response to high‐calorie foods (Goldstone *et al*., 2009). Perhaps more intriguingly, activation of brain reward systems in response to low‐calorie foods often appeared to decrease with fasting (Goldstone *et al*., 2009), paralleling the distinct lack of interest in non‐caloric foods displayed by PWS^*ICdel*^ mice. Interestingly, this may also tie‐in with endocrine changes seen in PWS, as artificially increased levels of ghrelin in healthy non‐obese subjects induced similar brain activation in response to food as fasting (Goldstone *et al*., 2014), and indeed to those seen in PWS (Miller *et al*., 2007; Dimitropoulos & Schultz, 2008). The idea for a role of ghrelin is supported by more direct evidence, showing that intracerebroventricular administration of ghrelin increased caloric food consumption without influencing LCS in rats (Overduin *et al*., 2012). However, other experimental studies are equivocal, with some showing that ghrelin enhances sweet taste food consumption and preference regardless of its caloric content (Disse *et al*., 2010); but others finding that ghrelin increases intake of rewarding food (Disse *et al*., 2010; Egecioglu *et al*., 2010). Consequently, these findings imply that the feeding abnormalities seen in PWS^*ICdel*^ mice are related to more than just hyperghrelinaemia alone.

The constant hunger state that has been attributed to PWS (Holland *et al*., 2003) could also offer a mechanism to the findings observed. A similar pattern of behaviour, normal hedonic response to but reduced intake of artificial sweetener, is seen in mice with artificially reduced glucose utilisation (Tellez *et al*., 2013). Here the suggestion is not that these animals are more sensitive to calories but, as sweet taste may guide ingestion by acting as a Pavlovian cue that signals ensuing metabolic consequences, glucoprivation removes a critical physiological signal involved in the control of goal‐directed sweetener intake (Tellez *et al*., 2013). Although not measured directly here, a Tg‐PWS mouse model, which shows global loss of PWS gene expression like the PWS^*ICdel*^ mice (Relkovic & Isles, 2013), has both hypoglycaemia (Stefan *et al*., 2005) and hypoinsulinaemia (Stefan *et al*., 2011). It is reasonable to suggest that the PWS^*ICdel*^ mice would display a similar metabolic phenotype, which in turn supports the idea that their altered basal physiology impacts on their response to saccharin. This account of the current data is also consistent with the observation that there was no difference between WT and PWS^*ICdel*^ mice in responding to water alone.

In conclusion, this study has examined food‐related behaviour in a mouse model for PWS, namely the PWS^*ICdel*^ mice. These animals show hyperphagia following overnight fasting, and also show increased free‐consumption of food made more palatable by the addition of sucrose. However, the hedonic response to consumption of sucrose solutions was completely normal, increasing with concentration as expected, but no larger (or smaller) than that seen in WT littermates. This suggests that the increased consumption and preference for high‐calorie foods such as sucrose and condensed milk seen here and in previous studies (Doe *et al*., 2009; Relkovic *et al*., 2010, 2012) is not due to increased hedonic value but an enhanced drive to pursue calorie‐rich foods. This idea is supported by the fact that PWS^*ICdel*^ mice seem to be particularly sensitive to the calorific content of palatable solutions, as indexed by the decrease in their responding to saccharin relative to WT controls. Taken together, and in the context of previous work with PWS subjects and other human experimental studies, these data point to the fact that feeding behaviour in PWS^*ICdel*^ mice, and possibly PWS generally, is primarily driven by changes in endocrine signalling to the hypothalamus that results in a neural state of almost constant hunger, which leads to enhanced calorie seeking and overeating.
